# Supplementary material for: Enhancing pigment production by a chromogenic bacterium (Exiguobacterium aurantiacum) using tomato waste extract: A statistical approach
Source: PLoS One. 2025 Jun 23;20(6):e0312922. doi: 10.1371/journal.pone.0312922 (PMC12184941; doi:10.1371/journal.pone.0312922)
Supplement: Attachment — Submitted filename: Response to Reviewers, MS-II, BZ.docx [file pone.0312922.s002.docx]

**Response to Reviewers’ comments**

It is our pleasure to hearing from the academic editor to submit the revised version of our manuscript considering its merit after addressing the points raised during the review process. We would like to thank the academic editor and reviewers for sparing their time in carefully going through the manuscript and providing scholarly comments in line with the journal requirements. Below are our point-by-point responses to your comments that we incorporated in the main manuscript.

**Response to journal requirements**

Requirement #1:

Ensuring manuscript meets PLOS ONE’S style requirements

Response to requirement #1:

We appreciate and accepted the given comments. The manuscript is now edited as per the PLOS ONE’S requirements including for file naming.

**Requirement #2:**

**Group authorship (wait for confirmation)**

Response to requirement #2

We appreciate your concern with regards to the authorship issue. The names of the authors are listed individually in order of their contribution in the acknowledgments section of the manuscript as per your request with their respective affiliations. The lead author of the manuscript is Birhanu Zeleke and his contact email address is:

E-mail: [zelekebirhanu4@gmail.com](mailto:zelekebirhanu4@gmail.com).

**Requirement #3:**

**About the phrase “data not shown” in the manuscript which does not meet your data sharing requirements**

Response to requirement #3

We acknowledge your observation. We mentioned it to indicate that we performed pair wise comparisons of Post hoc analysis to see significant differences among variables in the mean culture growth. The analysis generated 45 combinations which we fear a bit bulky to show all these data in the manuscript and may not attract readers. Therefore, as all the relevant information explaining about which AWEs have significant differences in mean culture growth within the manuscript, the phrase “data not shown” is removed from the manuscript.

**Requirement #4:**

Ensuring about completeness and correctness of reference list and citation of retracted articles

Response to requirement #4

We strongly acknowledge your concern and thoroughly revised our references for completeness and correctness using Crossref Metadata search and incorporated in the manuscript. With regards to citation of retracted paper, we tried to check all our references but did not come across with a retraction notice or any announcement about our references from the publisher’s website. Moreover, we have not received any alert from Zotero, the citation manager we have used for reference citation.
