# Supplementary material for: Enhancing pigment production by a chromogenic bacterium (Exiguobacterium aurantiacum) using tomato waste extract: A statistical approach
Source: PLoS One. 2025 Jun 23;20(6):e0312922. doi: 10.1371/journal.pone.0312922 (PMC12184941; doi:10.1371/journal.pone.0312922)
Supplement: Attachment — Submitted filename: Response to Reviewers.docx [file pone.0312922.s003.docx]

**Response to Reviewers**

It is our pleasure to receiving the message from the academic editor to submit the revised version of our manuscript considering its merit. We would like to thank the academic editor and reviewers for sparing their time in carefully reviewing the manuscript and providing us with scholarly comments. Below are our point-by-point responses to your points raised during the review process.

Point #1:

The manuscript describes about the use of natural pigment using TWE but the conclusion needs to be comprehensive.

Response to point #1:

We thank you for your valuable comment and fully accepted the raised point. Hence, the comment is well taken and the conclusion is now written in an elaborated way demonstrating the potential of utilizing agro waste extracts for bacterial cultivation as a cost-effective and nutritious substrate for the cultivation of pigment producing bacterial isolate, *Exiguobacterium aurantiacum*.

Point #2:

Please justify how the use of two statistical methods will help when this study is taken for scale up levels.

Response to point #2

We appreciate your point asking to justify how the uses of the two statistical methods help for scale up study. Below is the justification in brief.

PBD is a screening method used to identify the most influential factors out of many variables. Hence, it helps in quickly determining which factors have the most significant impact on the outcome, reducing the number of experiments needed by focusing only on the most important variables and ensuring that the scale-up process focuses on optimizing the critical factors identified, leading to more efficient and effective scaling.

Whereas as RSM is a collection of mathematical and statistical techniques used for optimizing processes, it helps in finding the optimal levels of multiple factors to achieve the desired response and understanding how different factors interact with each other and their combined effect on the outcome. Using the developed models, it is also used to predict outcomes under different conditions, ensuring an effective and efficient transition when laboratory study is taken for scale up levels.

Point #3:

In the abstract it is finally given that this study was done using AWE but in contrast the conclusion part says TWE is better-please justify.

Response to point #3

We acknowledge your question and below are our justification. This study began by evaluating various agro-waste extracts (AWEs) to determine which best supports the growth of the chromogenic bacterial isolate (*Exiguobacterium aurantiacum*). The study indicated that agro-waste decoctions can be viable substrates for bacterial growth. Notably, tomato waste extract (TWE) showed higher optical density (OD_600_) values, indicating better growth compared to other AWEs, and its performance was comparable to that of nutrient broth. ANOVA analysis confirmed that differences in OD values among the various AWEs were statistically significant, suggesting that TWE promotes superior bacterial growth. Consequently, TWE was concluded to be more effective in supporting the bacterial culture's growth compared to other tested AWEs for this study.

Point #4:

The manuscript should be checked for language corrections.

Response to point #4

We strongly accept and share your concern. Therefore, we thoroughly revised our manuscript for grammar and language corrections to our best.
