# Supplementary material for: Enhancing pigment production by a chromogenic bacterium (Exiguobacterium aurantiacum) using tomato waste extract: A statistical approach
Source: PLoS One. 2025 Jun 23;20(6):e0312922. doi: 10.1371/journal.pone.0312922 (PMC12184941; doi:10.1371/journal.pone.0312922)
Supplement: Attachment — Submitted filename: Response to Reviewers 22 May 2025.docx [file pone.0312922.s004.docx]

**PLOS ONE**

**Response to Reviewers**

It is our pleasure to receiving the academic editor's message regarding the submission of our revised manuscript, recognizing its merit. We sincerely appreciate the time and effort the academic editor and reviewers have dedicated to thoroughly evaluating our work and providing valuable feedback. Below, we present our point-by-point responses to each comment raised during the review process. Improvements and justifications have been integrated into the revised manuscript where applicable with yellow highlights indicating the changes for easy reference.

**Issues raised by the reviewer #1 and our response to each point-by-point:**

1. Language of the article needs improvement. There are several misleading statements such as in the line 70.

We have addressed the reviewer’s feedback by refining the manuscript's language to enhance clarity and overall quality. This includes correcting misleading statements, such as the one in line 70, as highlighted by the reviewer. All suggested improvements have been incorporated into the revised manuscript.

2. Abstract- should be improved to state key findings

The comment has been duly considered, and the abstract has been revised accordingly to incorporate the key findings as suggested. These improvements have been integrated into the revised manuscript.

3. In text citation should be corrected

All in-text citations have been carefully reviewed and corrected to ensure accuracy and consistency throughout the manuscript.

4. Whenever write OD, mention wavelength in subscript else it will remain unclear to the readers

The comment has been carefully considered, and the necessary corrections have been incorporated into the revised manuscript.

5. L152-154: Write about inoculum preparation and its standardization.

Acknowledging the valuable comment given by the reviewer, we included the inoculum preparation method and its standardization in the revised manuscript. The inoculum of the potent isolate (coded as PPPI-6) was prepared using the shake flask culture method, where a loopful of culture was transferred to nutrient broth and incubated over night at 30^o^C for activation. A pre-culture of 10 mL one-day-old culture suspension of the isolate was cultivated in each agro-waste extracts (AWEs) to assess their effectiveness in supporting culture growth. Growth was monitored daily by measuring optical density (OD_600_) until the stationary phase. At the stationary phase, the OD values of each broth culture were computed and statistically analyzed to identify the most nutritious AWEs, enhancing growth compared to the nutrient broth control.

Standardization was employed using Plackett-Burman Design (PBD) to screen significant factors affecting pigment production. Response Surface Methodology (RSM) was used to fine-tune those significant factors for maximum pigment yield, and statistical models predicted the best conditions for enhanced culture growth and pigment production using best AWE that support the culture growth as an alternative low-cost substrate. All experiments were performed in triplicates, with average values used for analysis.

6. Section 2.6: There isn’t any characterization given here. It is merely identification and elucidation of its structure

We appreciate the given comment and here below is our explanation to the raised question. The extracted pigments were analyzed to establish its comprehensive profile focusing on chemical composition, spectroscopic properties, and chromatographic separation for identification and structural elucidation. Chemical composition analysis, including compound classification, functional groups, and molecular structures were carried out using analytical methods. Spectroscopic properties, particularly absorption spectra for chromophore identification were examined via UV-Visible spectrophotometry. Functional groups and molecular interactions were further analyzed using ATR-FTIR. For chromatographic separation and ionization to determine its molecular weight and fragmentation pattern, LC-MS mass spectrometry was employed.

These methods collectively provide a thorough characterization of the extracted pigment, helping optimize its production, stability, and potential applications across various industries. The described explanation has been incorporated into the revised manuscript.

7. 3.1: Describe why this isolate was selected

The comment has been carefully considered, and the isolate was selected based on several key criteria. These include non-pathogenicity, confirmed through literature review, rapid growth in the chosen agro-waste extract, better pigment yield compared to other recovered pigment-producing isolates, and pigment stability under various environmental conditions in repeated laboratory trials. This explanation has been incorporated into the revised manuscript.

8. Improve presentation of the table 3.

The comment has been carefully addressed, and the table's presentation has been refined in the revised manuscript to enhance clarity and readability.

9. Table 8 and 9: Why OD values are given here when it is considered as a raw data

The reviewer’s observations have been thoroughly addressed by providing a detailed explanation of the analyzed data in both tables. Table 8 presents the initial experimental data, summarizing mean OD_600 nm_ values, biomass, and pigment yield across different combinations of independent variables to highlight their interaction effects. Table 9, on the other hand, focuses on the optimization process, summarizing ANOVA results to assess the significance and influence of each factor using statistical models. These include *F*-values, P-values, and R², which help evaluate model accuracy and the effect of individual factors. The clarifications have been incorporated into the revised manuscript.

10. Table 13: Why run 5 was not selected when it gives better results

We appreciate the reviewer’s insightful question. In Response Surface Methodology (RSM), the selection of optimal conditions is based on overall desirability, rather than solely focusing on the highest yield value. Although Run 5 shows better yield, the software takes a comprehensive approach, balancing yield, stability, reproducibility, and efficiency to ensure a reliable optimization process. The regression model in RSM further evaluates confidence intervals and error margins, leading to the selection of conditions that prioritize **c**onsistency over extreme values and ensure that the chosen combination aligns well with the mathematical model, producing reproducible and stable results.

11. Fig. 6: Standard deviation is missing

The comment has been duly acknowledged, and the standard deviation has been integrated into the graph in the revised manuscript to enhance data clarity and reliability.

12. L521: State which figure has been referred here

The figure is referring to the growth dynamics of *Exiguobacterium aurantiacum*, represented as a smooth line graph that tracks optical density (OD_600 nm_) variations over time.

13. Table 14: What is optimization efficiency?

Optimization efficiency refers to the percentage improvement achieved in the optimized values of outcome variables (OD_600 nm_ value, cell biomass, and pigment yield) compared to their initial un-optimized values, demonstrating the degree of improvement resulting from the applied optimization techniques.

**Issues raised by reviewer #2:**

Specific Comments:

1. Justification for selecting the specific variables for the PBD: Why these nine variables initially? Are they based on prior knowledge or literature?

2. Briefly explain the rationale behind selecting those specific ranges/levels for the independent variables in both the PBD and RSM designs.

**Response to the issues raised by the reviewer #2:**

We appreciate the reviewer’s thoughtful comments and we provide the following detailed justification for selecting specific variables for the PBD and the rationale behind the selection of those specific ranges for the independent variables in both the PBD and RSM designs.

Justification for selecting variables for PBD:

The selection of these variables and ranges was a data-driven process, combining literature reviews and preliminary laboratory experiments. When optimizing pigment production in bacterial isolates, various environmental and nutritional factors directly influence their metabolism, growth dynamics, and biosynthesis efficiency.

In our case, the nine independent variables were selected for PBD screening and identified based on comprehensive literature review as well as empirical laboratory trials. Previous studies were thoroughly reviewed on factors affecting bacterial growth and pigment biosynthesis which provided us basis for selecting these parameters. We also performed repeated laboratory based one-variable-at-a-time experiments for eco-physiological tolerance which helped us refine the choice of important factors affecting growth rate and pigment yield.

Rationale for selecting specific ranges for PBD & RSM:

The range selection for independent variables in both PBD and RSM designs were also guided by prior knowledge from existing literature review and repeated experimental trials for eco-physiological tolerance. Prior knowledge from earlier studies on bacterial pigment production provided us insights into typical optimal conditions. Repeated experimental trials from tracking the culture growth at OD_600 nm_ at different variable levels also allowed us to estimate conditions best support maximum bacterial growth.

From preliminary screening results, initial laboratory trial tests identified threshold values for important environmental parameters, ensuring that the selected ranges cover both minimum and maximum feasible levels. Finally, PBD was used to screen significant factors, while RSM further fine-tuned the most impactful variables to maximize pigment yield.

Best regards,
